# Multi-parameter vital sign database to assist in alarm optimization for general care units

**DOI:** 10.1007/s10877-015-9790-8

**Published:** 2015-10-06

**Authors:** James Welch, Benjamin Kanter, Brooke Skora, Scott McCombie, Isaac Henry, Devin McCombie, Rosemary Kennedy, Babs Soller

**Affiliations:** Sotera Wireless Inc., 10020 Huennekens St., San Diego, CA 92121 USA

**Keywords:** Patient monitoring, Vital sign management, Alarm fatigue, Rapid response system

## Abstract

Continual vital sign assessment on the general care, medical-surgical floor is expected to provide early indication of patient deterioration and increase the effectiveness of rapid response teams. However, there is concern that continual, multi-parameter vital sign monitoring will produce alarm fatigue. The objective of this study was the development of a methodology to help care teams optimize alarm settings. An on-body wireless monitoring system was used to continually assess heart rate, respiratory rate, SpO_2_ and noninvasive blood pressure in the general ward of ten hospitals between April 1, 2014 and January 19, 2015. These data, 94,575 h for 3430 patients are contained in a large database, accessible with cloud computing tools. Simulation scenarios assessed the total alarm rate as a function of threshold and annunciation delay (s). The total alarm rate of ten alarms/patient/day predicted from the cloud-hosted database was the same as the total alarm rate for a 10 day evaluation (1550 h for 36 patients) in an independent hospital. Plots of vital sign distributions in the cloud-hosted database were similar to other large databases published by different authors. The cloud-hosted database can be used to run simulations for various alarm thresholds and annunciation delays to predict the total alarm burden experienced by nursing staff. This methodology might, in the future, be used to help reduce alarm fatigue without sacrificing the ability to continually monitor all vital signs.

**Electronic supplementary material:**

The online version of this article (doi:10.1007/s10877-015-9790-8) contains supplementary material, which is available to authorized users.

## Background

In 2004 the Institute for Healthcare Improvement (IHI) launched the “100,000 Lives Campaign” to significantly reduce morbidity and mortality in the US healthcare system. Six steps were identified, one of which was to deploy rapid response teams at the first sign of patient decline. In 2006, with the initial goal exceeded, IHI introduced a “5 Million Lives Campaign” again recommending rapid response teams as a core component recognizing that ‘failure to rescue’ events are a major cause of mortality in American hospitals.

An international consensus conference suggested that to reduce failure to rescue events a rapid response system (RRS) is necessary. A RRS is composed of four components: an afferent limb (detection and response triggers), an “efferent limb” (technical and human resources brought to the bedside), a quality limb, and an administrative limb [1]. Studies investigating in-hospital mortality have shown deterioration of vital signs 6–12 h prior to a serious clinical event [2–4]. Yet, despite rising inpatient acuity levels, the standard for routine physiological assessment outside the ICU is once every 4–8 h. The conference identified a core set of parameters which should be continually monitored for the goal of early detection of physiological instability: heart rate (HR), blood pressure, respiratory rate, temperature, pulse oximetry, and level of consciousness [1].

One of the concerns about implementing multi-parameter continual vital sign monitoring on a general medical or medical-surgical unit is alarm fatigue. The high rate of alarms, and their sometimes limited clinical relevance has been well documented in the operating room [5] and ICU [6, 7] and the concern about alarm fatigue is real [7]. In general medical units, the nurse to patient ratio is lower than in the OR and ICU, so continual monitoring in this environment has the potential for being a significant burden on nurses.

Several methods have been evaluated to reduce the number of non-actionable alarms during continual monitoring of patients. One successful approach is to combine alarm thresholds with annunciation delays (a delay between when an alarm threshold has been crossed and when the alert is sounded or displayed) [8–12]. In most cases alarm thresholds are set based upon knowledge of and experience with the vital sign measures. Recently, work has been done to develop evidence-based methods for determining alarm limits. Burgess et al. [11] established a database of HR and respiration rate (RR) measurements from 317 patients (18,737 h) in a general care unit, with no adverse events. Modeling was done with this database to predict the alarm rate for different alarm limits, with the goal of reducing the number of false positive alarms. Welch [12] describes a method for reducing SpO_2_ alarms based on creating a large database of measurements. The database is used to predict the alarm rate based upon combinations of alarm thresholds and annunciation delays. The current study extends Welch’s methodology [12] to also include HR, RR, systolic, diastolic and mean blood pressure (SBP, DBP, MAP), by evaluating a method for optimizing alarm rates for continual multi-parameter monitoring in general care units.

## Methods

### Vital sign data collection

Vital sign data were collected throughout the day and night with the ViSi Mobile System, an on-body, multi-parameter monitoring platform capable of continual measurement and display of core vital signs including ECG, RR, HR, continual NIBP (cNIBP), pulse oximetry (SpO_2_), pulse rate, and skin temperature (Sotera Wireless Inc., San Diego, CA, USA) [13]. Data capture included waveforms (500 samples/s) and numeric data displayed as a 3 s moving average.

The ViSi Mobile System transmitted patient data via the existing wireless network in the hospital. De-identified patient data were uploaded through a secure link to a private cloud at the end of each patient’s monitoring session. The numeric values in the cloud-hosted database were then used to simulate the total alarm burden associated with simultaneous monitoring of all key vital signs.

### Alarm rate simulation and independent hospital evaluation

A range of potential alarm thresholds were chosen by the authors and used to model the impact of annunciation delays on the resulting total alarm rate. Ranges for the alarm thresholds used in the simulations were chosen based upon the distribution of vital sign values in the database (Supplemental Data). Adverse events in general care units are rare, so in a large population it can be assumed that alarm thresholds should be set near the tail ends of each distribution. Tables were constructed for each vital sign showing the projected number of alarms for each combination of threshold and annunciation delay. Specific alarm thresholds and annunciation delays were selected for each vital sign and the total alarm rates (number of alarms/patient/day) were calculated. A 10 day evaluation was conducted at an independent hospital, not included in the cloud-hosted database, over the period January 20, 2015–January 30, 2015. Continual SpO_2_, HR, RR, cNIBP data were collected for these patients and alarm rates were calculated using the same thresholds and annunciation delays as above. The alarm rate calculated for the independent hospital was compared with the simulation done with the data in the cloud-hosted database.

### Database comparisons

**Fig. 1:**
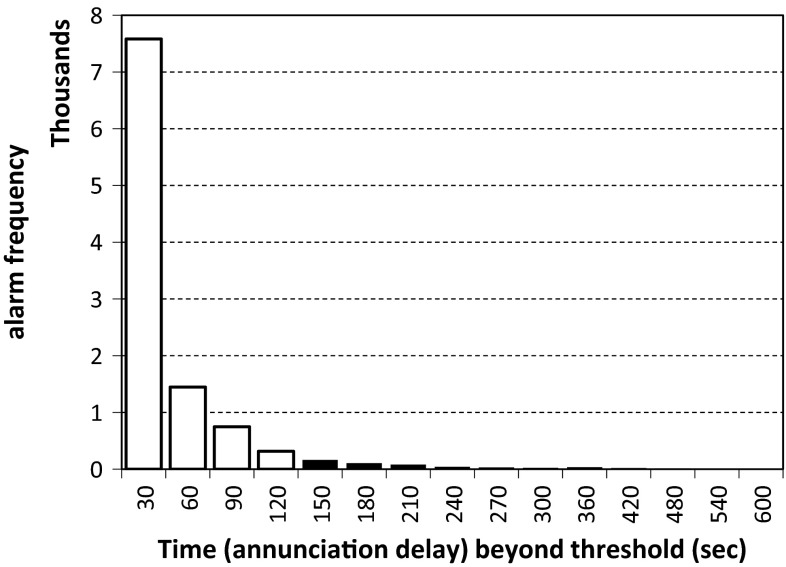
Example of the effect of annunciation delay on the alarm rate for the high respiration rate threshold; 63,074 h of data from 1919 patients at a single hospital over 1 year. *White bars* represent alarms suppressed by setting the delay at 120 s

Since alarm threshold values were determined from the population histograms of data in the cloud-hosted database, we assessed general applicability of the method by comparing these distributions to data from previously published databases. One comparison was made with the 1.15 million individual vital sign determinations from 27,722 patients reported by Bleyer et al. [14], collected intermittently for patients on all non-intensive care unit and all non-intermediate care unit floors at a single institution. The data to re-plot the histograms for the comparison were obtained from the online supplement [14]. A second comparison was made to data from Tarassenko et al. [15]; 64,622 h of vital sign measurements from 863 patients. These data were collected from continual five-parameter monitoring in med-surg patients at one hospital in the US and one hospital in the UK. To re-plot the histograms the data were read digitally from Figure 1 in the Tarassenko paper [15] using WebPlotDigitizer Version 3.4 (A. Rohatgi 2014, http://arohatgi.info/WebPlotDigitizer). For comparison, all histograms were normalized so the total area under the curve was 1.

## Results

### Cloud-hosted database

The cloud-hosted database analyzed in this paper is composed of 94,575 h of monitoring data for 3430 patients. Table 1 is an example of the simulation for low SpO_2_ alarm. Each cell within the table shows the predicted number of alarms for the corresponding threshold setting and annunciation delay value. This simulation is centered on an alarm limit of 85 % with a delay of 30 s; with this particular setting one can expect 3.7 SpO_2_ alarms/patient/day. By examining all of the cells, the relative impact of changing alarm threshold or annunciation delay can be seen. As an example, at a threshold value of 85 %, increasing the delay from 20 to 40 s, leads to a 44 % reduction in the number of expected alarms. Abbreviated tables for the other vital signs can be found online as supplemental data.

**Table 1:** Projected alarm rates (number of alarms/patient/day) for a low SpO_2_ alarm as a function of SpO_2_ threshold and annunciation delay

	Threshold—low SpO_2_				
	81	83	85	87	89
Annunciation delay (s)					
20	0.9	1.9	4.8	12.3	28.8
25	0.8	1.6	4.0	10.4	24.5
30	0.7	1.5	3.7	9.6	22.8
35	0.6	1.3	3.1	8.3	19.8
40	0.6	1.1	2.7	7.2	17.5

Tables for the other vital signs are shown in the data supplement

Figure 1 shows an example of the effect of annunciation delay on the alarm rate for the high RR alarm. The white bars represent alarms suppressed by setting the delay at 120 s. Seventy-one percent of the alarms would last 30 s or less; 95 % of the alarms resolve by themselves within 120 s.

Alarm threshold and annunciation delays were selected for each vital sign and are shown in Table 2. These settings were then applied to the cloud-hosted data to calculate the overall alarm burden that would be experienced by a nurse using all of the vital signs to monitor a patient. Table 3 shows the individual vital sign and total alarm rates that can be expected if these specific alarm settings are adopted. The total alarm rate based upon that particular group of settings is projected to be 10.3 alarms/patient/day, with SpO_2_ and HR each contributing about 40 % of the alerts.

**Table 2:** The alarm settings used to simulate total alarm burden

	High threshold	Annunciation delay for high threshold	Low threshold	Annunciation delay for low threshold
Heart rate	150 beats/min	5 s	30 beats/min	5 s
Respiration rate	35 breaths/min	120 s	4 breaths/min	120 s
SpO_2_	N/A	N/A	85 %	30 s
Systolic BP	190 mmHg	60 s	N/A	N/A
Diastolic BP	N/A	N/A	N/A	N/A
Mean arterial pressure	N/A	N/A	60 mmHg	60 s

*N/A* not used in calculating total alarm rate

**Table 3:** Total alarm burden projected from the cloud-hosted database and the independent test hospital 10 day evaluation for each vital sign and the aggregate

	Cloud-hosted database alarm rate (alarms/patient/day)	Independent hospital alarm rate (alarms/patient/day)
Heart rate	4.2	1.2
Respiration rate	0.6	0.6
SpO_2_	3.7	1.3
cNIBP	1.9	7.6
Total	10.3	10.6

### Independent hospital evaluation

A total of 1550 h of monitoring data were collected for 36 patients during a 10 day evaluation in an independent hospital whose data were not included in the cloud-hosted database. The total alarm rate (10.6 alarms/patient/day) for the patients in the independent hospital was calculated using the same settings and is comparable to the total alarm rate calculated from the full database (Table 3), however, for the independent hospital data, cNIBP contributes to the largest number of alarms to the total.

### Database comparisons

**Fig. 2:**
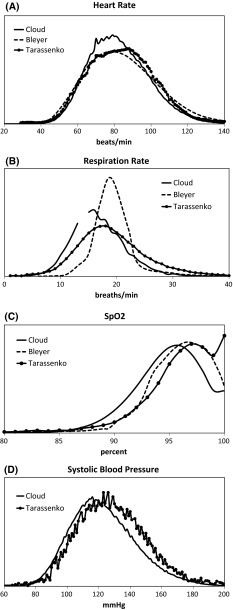
Comparison of cloud-hosted vital sign data with previously published data collected from general care and medical-surgical units. Cloud-hosted database: continual data collection; 94,575 h, 3430 patients. Tarassenko database (14): continual data collection; 64,622 h, 863 patients; Bleyer database (13): intermittent data collection; 1.15 million individual determinations from 27,722 patients. Total area under each curve was normalized to 1

Figure 2 shows the comparison of the cloud-hosted database to the other two published studies [14, 15] for HR, RR, SpO_2_ and SBP. Data for RR of 14 in the cloud-hosted database and SBP between 100 and 160 mmHg in the Bleyer data were missing and not included in figure.

Table 4 reports the mean and 95 % confidence intervals for the three datasets. Because the data consist of repeated measures on fewer patients than the number of measurements and the correlation between repeated measures is unknown, we conservatively used the number of patients as the degrees of freedom in confidence interval calculations. Differences between the means for each of the parameters are small and clinically insignificant. Table 4 also shows the 1, 5, 10, 90, 95 and 99 % percentile values where available. The distribution extremes (1, 5, 95 and 99 %) have been used by others to identify patients at risk for the development of early warning scoring systems [15]. The extreme values determined from the cloud database agree well with both databases for SpO_2_ and with Tarassenko’s results [15] for HR, RR and SBP.

**Table 4:** Distribution of vital sign measurements from the cloud-hosted database, the Bleyer et al. [14] and the Tarassenko et al. [15] studies

	1 %	5 %	10 %	Mean	Median	90 %	95 %	99 %
Heart rate								
Cloud	50	58	63	82.7 (82.1–83.2)	81	105	112	128
Bleyer	45	55	65	84.7 (84.5–84.9)	85	115	125	145
Tarassenko (paper)	50	58	63	84.2 (83.0–85.4)	N/A	105	113	128
Respiration rate								
Cloud	7	10	11	16.8 (16.7–17.0)	16	23	25	30
Bleyer	13	15	15	19.7 (19.6–19.7)	19	22	24	34
Tarassenko (paper)	7	10	13	18.6 (18.2–19.0)	N/A	26	29	34
SpO_2_								
Cloud	86	89	91	94.9 (94.8–95.0)	95	99	100	100
Bleyer	86	91	96	96.0 (95.9–96.0)	97	100	100	100
Tarassenko (paper)	84	90	93	96.0 (95.8–96.2)	N/A	N/A	N/A	N/A
Systolic blood pressure								
Cloud	83	93	98	123.4 (122.7–124.1)	121	151	161	180
Tarassenko (paper)	85	96	101	128.5 (127.1–129.9)	N/A	155	165	185

Mean and 95 % confidence interval reported

*N/A* data not available, SBP data missing for Bleyer

## Discussion

This study demonstrated the use of a large database for simulating the effect of alarm thresholds and annunciation delays on the total alarm burden we anticipate might be experienced by nurses doing multi-parameter patient monitoring in a general care unit. The cloud-hosted database was used to perform numerous simulations over a large range of alarm thresholds and annunciation delays. When the total alarm burden was estimated using specific threshold and annunciation delays for each vital sign the same total alarm rate was found for both the large database and the independent test hospital, indicating that the cloud-hosted database is a good representation of the patient population in a general care unit.

Low alarm rates can be achieved by selecting thresholds that represent the lowest and highest 0.5–1.0 % of each vital sign distribution (Table 4) and choosing annunciation delays that suppress alerts that resolve themselves quickly (Table 2; Fig. 1). We did not assess if the suppressed alarms were indeed actionable clinical events, but including an annunciation delay is known to eliminate transient and motion artifacts which can be a major source of false alarms [10].

It has been estimated that there are between 100 and 200 alarms/patient/day in the ICU [6, 7]. Patients in general care units are presumed to be more stable, and therefore would be expected to have a lower alarm rate. Taenzer et al. [10] report four alarms/patient/day using a SpO_2_-only system. Our results, using a different pulse oximeter demonstrated similar results (3.7 alarms/patient/day for SpO_2_). In our study we demonstrate that it is feasible, though not necessarily sufficient, to use population data to assess the impact of alarm thresholds and annunciation delays for all vital signs, giving clinicians control over the number of alerts generated by monitoring systems.

Figure 2 and Table 4 demonstrate that the distribution of vital signs for the general care unit is independent of the monitoring equipment used to make the measurement. Comparison of individual patient values to the distribution may provide guidance to the nurse and aid in their decision making process. Values significantly outside the expected distribution could indicate patient deterioration, equipment malfunction or a patient with unusual physiology. Further patient assessment might lead to the appropriate action: therapy, equipment adjustments (replace dislodged nasal cannula, for example) or individualized alarm settings, respectively for the causes listed above.

### Limitations

This study demonstrated a data-driven methodology for managing the number of alarms when multi-parameter monitoring is done in general care units. While the methodology is generalizable, the results in Fig. 2 and Table 4 and the supplement are specific to general care units and would not be applicable for continual monitoring in the ICU or the OR. The specific values for alarm thresholds and annunciation delays have been determined for continual vital sign monitoring and may not be appropriate for spot check monitoring for general care units, where intermittent monitoring may require higher sensitivity.

The cloud-hosted database represents ten hospitals with data collection over a 9 month period. cNIBP data became more prevalent during the later periods of data collection, hence the percentage of patients with cNIBP measurements in the independent hospital was higher than in the overall database. It is likely that this observation explains the higher rate of cNIBP alarms in the test hospital data. The database continues to grow and is expected in the future to be more representative in predicting the number of cNIBP alarms. Similarly, during the course of the study we made algorithm improvements to the pulse oximeter sensor which lowered the alarm rate for SpO_2_ and HR for the independent hospital.

This study described one way data can be used to manage alarms and alerts during continual multi-parameter monitoring in general care units. This is in contrast to the ICU setting where there are a large number of patient adverse events and alarm thresholds are optimized for enhanced sensitivity to these events. The work described here was not designed to demonstrate sensitivity of the alarm settings to actionable clinical events, nor was it designed to compare this methodology to other methods of using vital sign data to alert nurses to patient deterioration.

## Conclusions

This study produced a large cloud-hosted database documenting the distribution of all standard vital sign measurements for 94,575 h of continual patient monitoring in general care units. The cloud-hosted database was used to demonstrate a methodology for selecting alarm thresholds and annunciation delays based on simulations of the total alarm burden that might be expected. The approach was validated with an independent dataset, demonstrating applicability to a new hospital. Similarities in the distribution of vital sign data in the cloud-hosted database with vital sign distributions previously published, suggests that this methodology could also be applied to data collected using other multi-parameter vital sign monitors. This effort is consistent with and directly supportive of the US Joint Commission National Patient Safety Goal 06.01.01.

## Electronic supplementary material

Below is the link to the electronic supplementary material.

Supplementary material 1 (PDF 976 kb)

Supplementary material 2 (PDF 65 kb)

Supplementary material 3 (PDF 63 kb)

Supplementary material 4 (PDF 64 kb)

Supplementary material 5 (PDF 51 kb)

Supplementary material 6 (PDF 62 kb)

Supplementary material 7 (PDF 65 kb)
