# Supplementary material for: Multi-parameter vital sign database to assist in alarm optimization for general care units
Source: J Clin Monit Comput. 2015 Oct 6;30(6):895–900. doi: 10.1007/s10877-015-9790-8 (PMC5081381; doi:10.1007/s10877-015-9790-8)
Supplement: Supplementary file 1 — Supplementary material 1 (PDF 976 kb) [file 10877_2015_9790_MOESM1_ESM.pdf]

Supplemental Data

Projected alarm rates (number of alarms/patient/day) from simulations in the cloud-hosted database

Threshold - Heart Rate

delay (s)	25	35	45	135	145	155
0	4.9	6.5	17.5	10.7	5.5	2.6
5	2.4	3.3	10.2	5.0	2.3	0.9
10	1.9	2.5	7.8	3.8	1.7	0.7
15	1.5	1.9	6.2	3.0	1.3	0.5

Threshold - Respiratory Rate

delay (s)	2	4	6	30	35	40
90	0.2	0.4	1.4	2.5	0.5	0.1
105	0.1	0.3	1.1	2.1	0.4	0.1
120	0.1	0.3	0.9	1.8	0.3	0.0
135	0.1	0.2	0.7	1.5	0.3	0.0
150	0.1	0.2	0.6	1.3	0.2	0.0

Threshold - SpO2

delay (s)	80	85	90
20	0.7	4.8	42.1
25	0.6	4.0	36.1
30	0.6	3.7	33.7
35	0.5	3.1	29.5
40	0.4	2.7	26.2

Threshold - cNIBP Systolic

delay (s)	180	190	200
30	2.7	1.2	0.6
45	2.3	1.1	0.5
60	2.0	0.9	0.4
75	1.8	0.8	0.4
90	1.5	0.7	0.3

Threshold - cNIBP - MAP

delay (s)	55	60	65
30	0.4	1.3	3.4
45	0.3	1.1	2.8
60	0.3	0.9	2.5
75	0.2	0.7	2.1
90	0.2	0.6	1.9
