# Supplementary figures and images for: Multi-parameter vital sign database to assist in alarm optimization for general care units
Source: J Clin Monit Comput. 2015 Oct 6;30(6):895–900. doi: 10.1007/s10877-015-9790-8 (PMC5081381; doi:10.1007/s10877-015-9790-8)

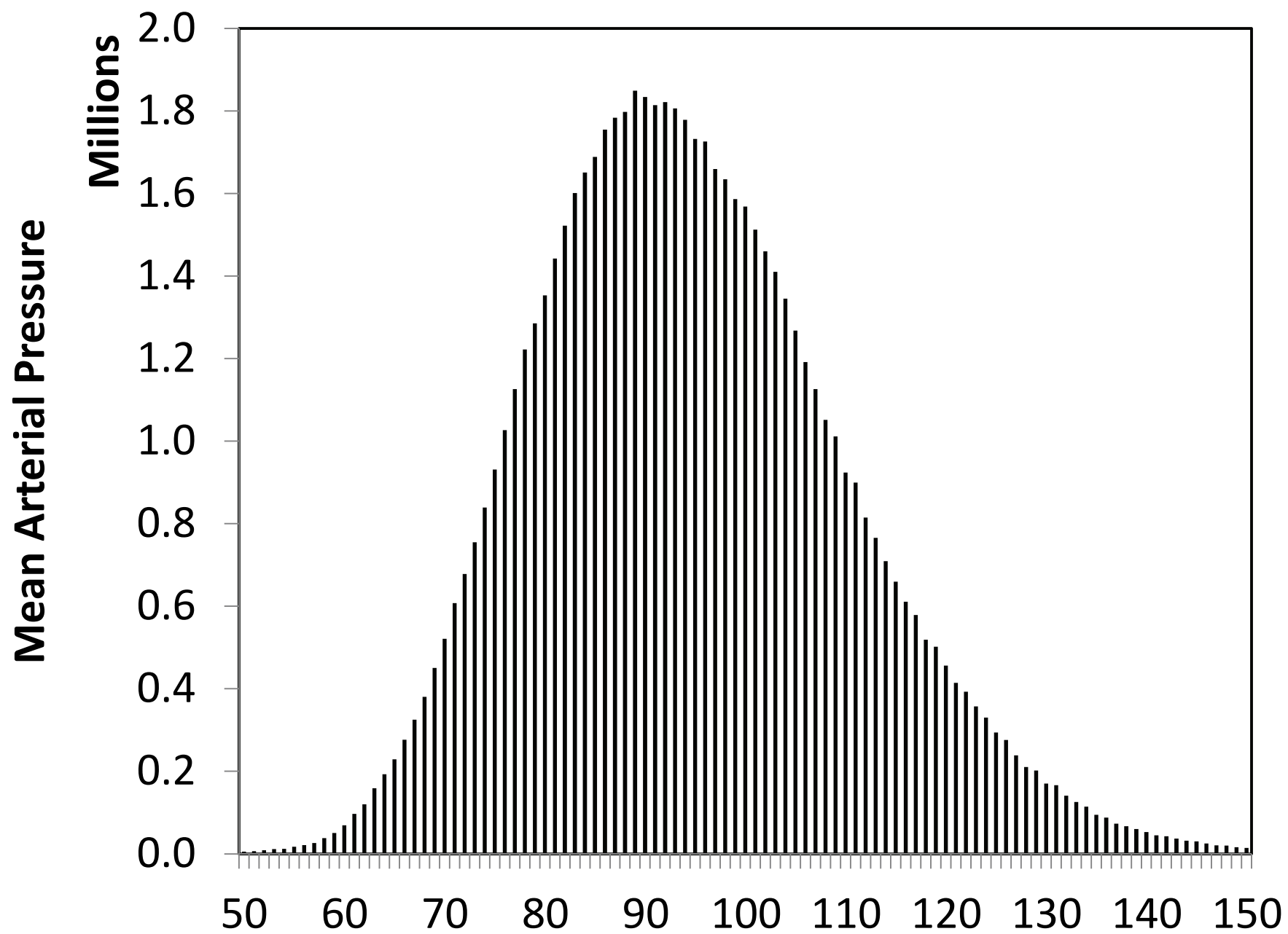

Supplement: Supplementary file 2 — Supplementary material 2 (PDF 65 kb) [file 10877_2015_9790_MOESM2_ESM.pdf]

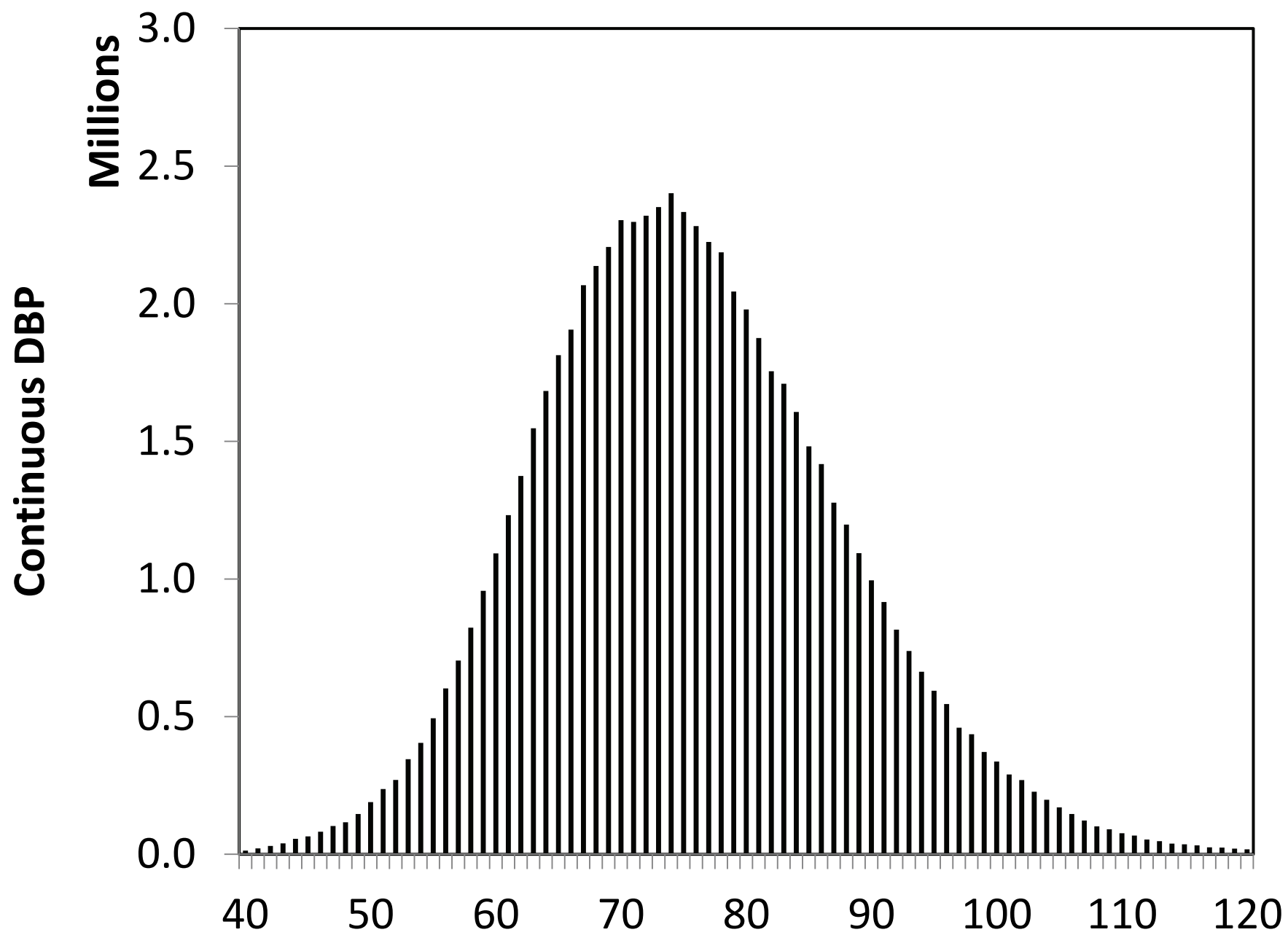

Supplement: Supplementary file 3 — Supplementary material 3 (PDF 63 kb) [file 10877_2015_9790_MOESM3_ESM.pdf]

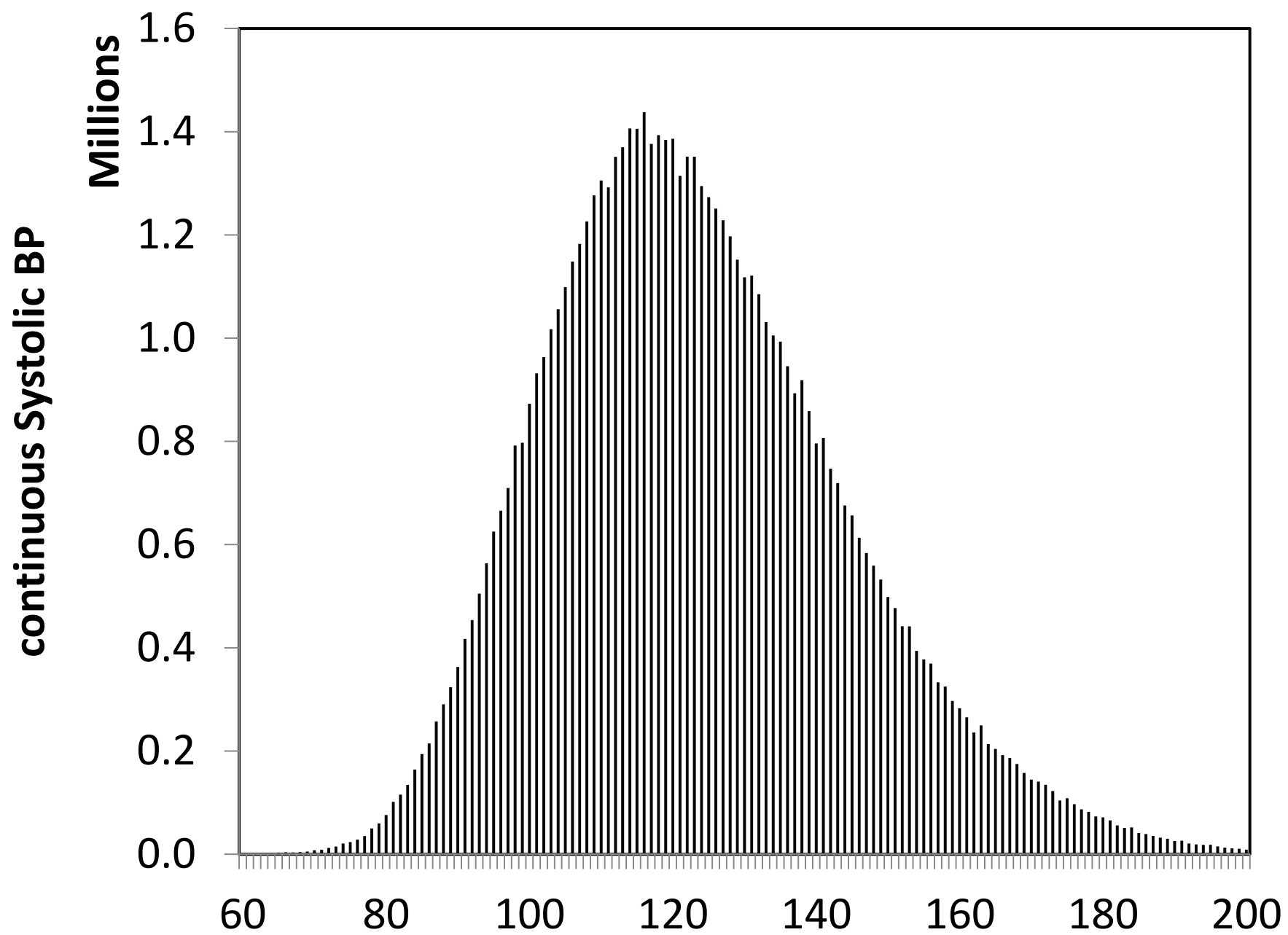

Supplement: Supplementary file 4 — Supplementary material 4 (PDF 64 kb) [file 10877_2015_9790_MOESM4_ESM.pdf]

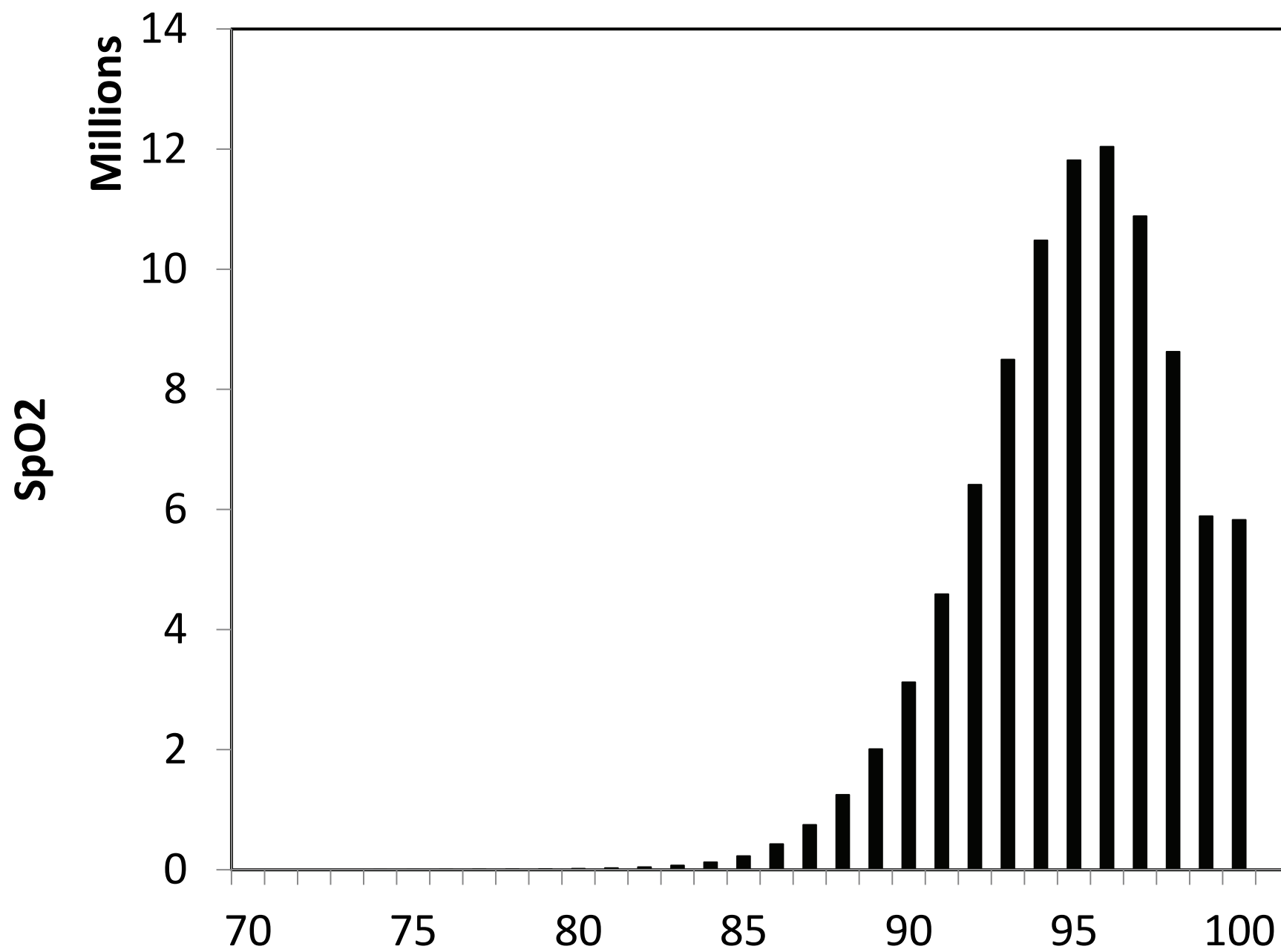

Supplement: Supplementary file 5 — Supplementary material 5 (PDF 51 kb) [file 10877_2015_9790_MOESM5_ESM.pdf]

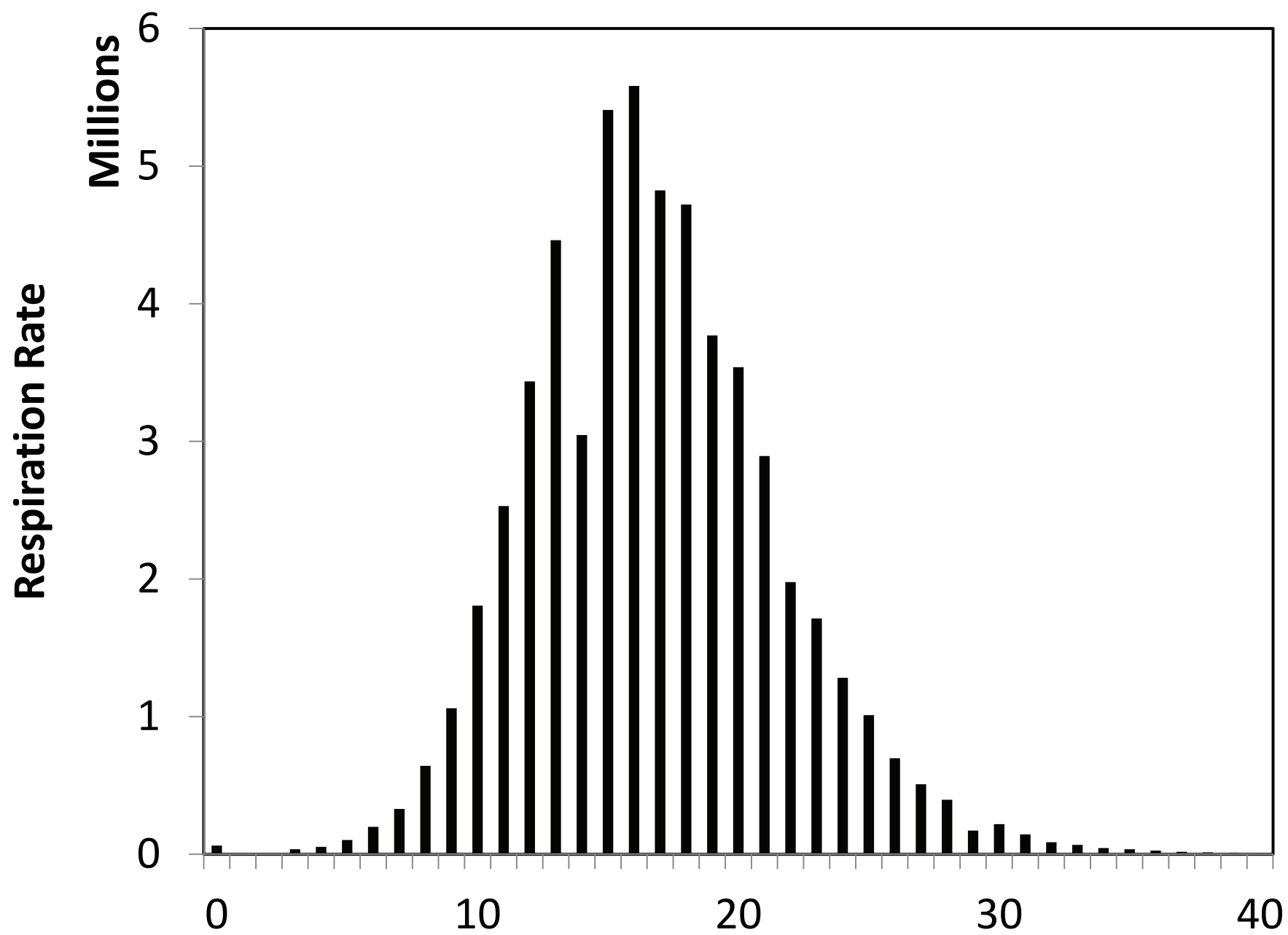

Supplement: Supplementary file 6 — Supplementary material 6 (PDF 62 kb) [file 10877_2015_9790_MOESM6_ESM.pdf]

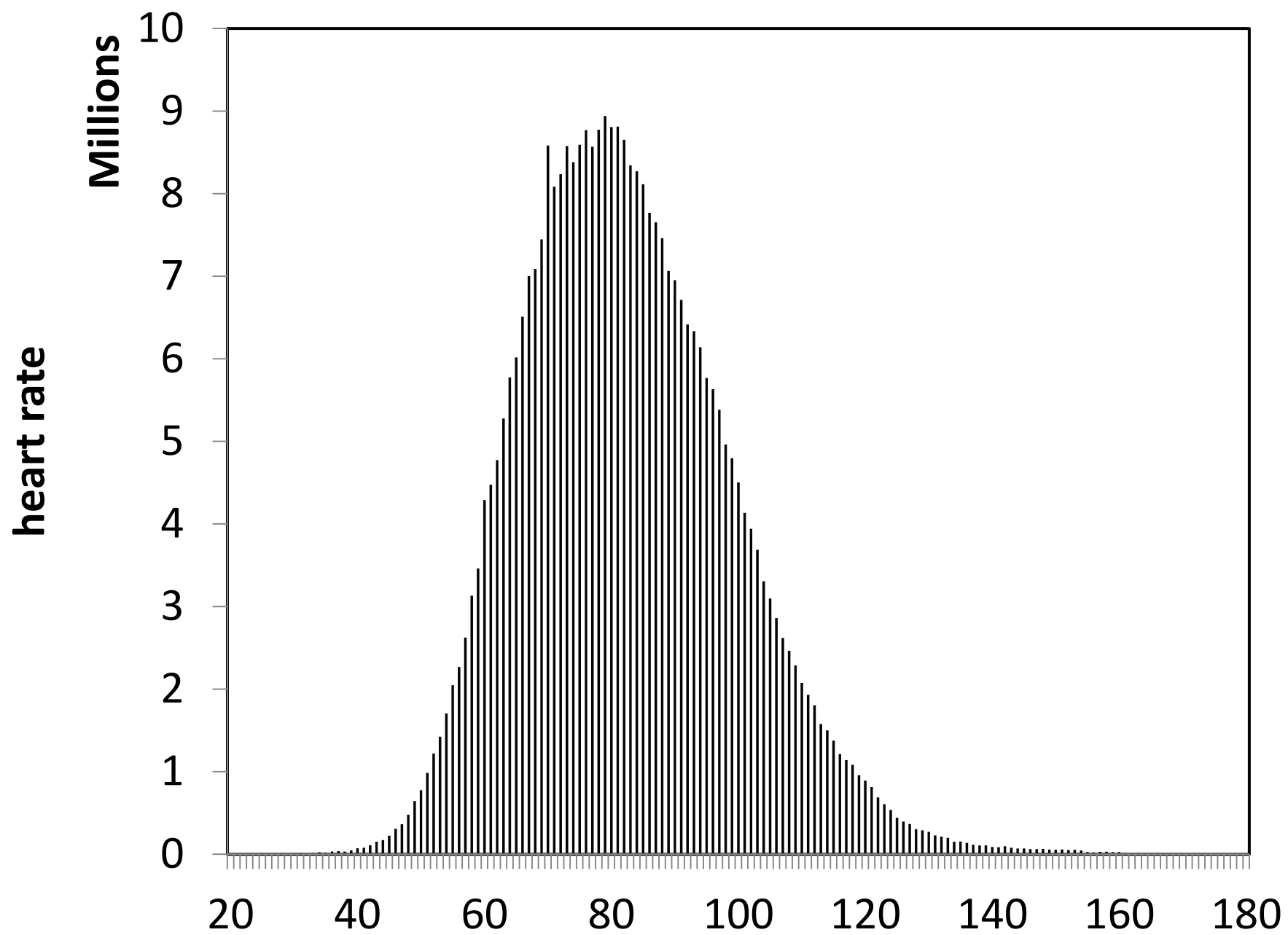

Supplement: Supplementary file 7 — Supplementary material 7 (PDF 65 kb) [file 10877_2015_9790_MOESM7_ESM.pdf]
